# Characteristic Evaluation of Recombinant MiSp/Poly(lactic-*co*-glycolic) Acid (PLGA) Nanofiber Scaffolds as Potential Scaffolds for Bone Tissue Engineering

**DOI:** 10.3390/ijms24021219

**Published:** 2023-01-07

**Authors:** Yuan Sun, Xiaona Jia, Qing Meng

**Affiliations:** College of Biological Science and Medical Engineering, Donghua University, Shanghai 201620, China

**Keywords:** recombinant spider silk proteins, PLGA, nanofibrous scaffolds, bone tissue engineering

## Abstract

Biomaterial-based nanofibrous scaffolds are the most effective alternative to bone transplantation therapy. Here, two recombinant minor ampullate spidroins (spider silk proteins), R1SR2 and NR1SR2C, were blended with Poly(lactic-co-glycolic) Acid (PLGA), respectively, to generate nanofiber scaffolds by electrospinning. The N-terminal (N), C-terminal (C), repeating (R1 and R2) and spacer (S) modules were all derived from the minor ampullate spidroins (MiSp). The physical properties and structures of the blended scaffolds were measured by scanning electron microscopy (SEM), water contact angle measurement, Fourier transform infrared spectroscopy (FTIR), Differential scanning calorimetry (DSC), and Tensile mechanical testing. The results showed that blending of MiSp (R1SR2 and NR1SR2C) reduced the diameter of nanofibers, increased the porosity and glass transition temperatures of nanofibrous scaffolds, and effectively improved the hydrophilicity and ultimate strain of scaffolds. It is worth noting that the above changes were more significant in the presence of the N- and C-termini of MiSp. In cell culture assays, human bone mesenchymal stem cells (HBMSCs) grown on NR1SR2C/PLGA (20/80) scaffolds displayed markedly enhanced proliferative and adhesive abilities compared with counterparts grown on pure PLGA scaffolds. Jointly, these findings indicated recombinant MiSp/PLGA, particularly NR1SR2C/PLGA (20/80) blend nanofibrous scaffolds, is promising for bone tissue engineering.

## 1. Introduction

Bone defects or dysfunction caused by trauma, congenital bone malformations and other disorders affect up to 20 million individuals each year [1,2,3]. Meanwhile, the prevalence of bone-related pathologies, e.g., bone cancer, osteoporosis, bone infection and osteoarthritis, are increasing year by year. Bone tissue has a limited ability to heal itself. Bone defects caused by tumor resection, cancer, severe mechanical injury, or genetic abnormalities often fail to heal on their own [4], resulting in a very high demand for the treatment of bone defects. Bone grafts and bone replacements are currently the routine treatment options applied for 2.2 million surgeries per year [5,6]. However, as an invasive surgery, the risk of immune rejection and cross infection caused by bone graft is often unavoidable after surgery [7,8]. Therefore, bone grafting and bone replacement surgery are limited in some situations. In order to solve the contradiction between the high demand for the treatment of bone tissue-related diseases and the high risk of conventional transplantation, the search for effective alternatives to bone transplantation has attracted increasing attention of medical professionals [9]. Scaffold implantation in bone tissue engineering promotes autologous bone regeneration, which may be an effective alternative to bone grafting [10,11,12].

Unlike permanent bone implants, scaffolds are implanted to provide effective temporary support for cell adhesion. Multiple reports have demonstrated nanostructured scaffolds are more efficient than macrosized scaffolds in promoting bone regeneration [7]. Because nanoscaffolds have large surface areas and high porosity, they artificially mimic the extracellular matrix to a certain extent, providing an excellent natural environment for tissue regeneration processes, including cell adhesion, proliferation and differentiation [13,14]. Common nanofiber fabrication protocols comprise phase separation, self-assembly [15], melt blowing [16] and electrospinning [17]. Phase separation is a method to induce the separation of homogeneous polymer solutions into two or multiphase system domains based on thermal energy changes [18]. Self-assembly (SA) systems that emulate ECM conditions and provide a platform for drug delivery have been used in neural tissue engineering [19]. Melt blowing refers to the process in which nanofiber nonwovens are attenuated by aerodynamic forces in turbulent flow fields. Of these, electrospinning, which was developed in the 1930s, with mature preparation technology, simple setup and operation method, has become an attractive scheme for the preparation of nanoscaffolds in biomedicine and tissue engineering [20,21,22]. Evidence suggests organic macromolecules, biomolecules, proteins, polymers, inorganic compounds, etc. can be blended into nanofibers by electrospinning under appropriate conditions [23,24,25,26,27].

PLGA is a biodegradable copolymer that has been widely used for the preparation of nanofiber scaffolds. PLGA has had approval from the U.S. Food and Drug Administration (FDA) for human clinical trials [28]. The material has the potential to interact with biological materials by changing surface properties [29]. It is one of the most commonly biodegradable polymers utilized for 3D scaffolds in tissue engineering [30,31]. However, despite some biocompatibility, pure PLGA showed suboptimal mechanical features in clinical applications of bone regeneration, hampered by biological and physical properties [32]. Therefore, PLGA is often used in combination with other materials [33,34,35] to enhance its mimicry and improve bone regeneration more effectively [36]. Spider silk protein is a biological macromolecular protein with superior performance [37,38]. Due to non-toxic nature, high biocompatibility, flexibility, elasticity, cell adsorption and growth enhancement, chemical modification and other properties, spider silk protein has been widely utilized in multiple biomedical engineering processes as a biological material in the form of thin film, 3D scaffold, electrospinning scaffold, hydrogel, microsphere and other forms [39,40,41,42]. It is a material with high reliability for bone tissue engineering [43]. In previous studies, minor ampullate silks showed quite good water solubility, elasticity and biocompatibility [44]; therefore, we speculated that the combination of secondary ampulla filaments and PLGA materials for electrospinning may yield nanoscaffold materials with better performance. To the best of our knowledge, there is no reported case study on the application of minor ampullate spidroins and PLGA mixed electrospinning nanofiber materials in bone tissue engineering.

Based on the above substantial information, this study adopted the electrospinning technology to prepare nanofiber scaffolds blended with minor ampullate spidroins R1SR2 and NR1SR2C and PLGA, respectively, and prepared pure PLGA nanofiber scaffolds as the control. scanning electron microscopy (SEM), water contact angle measurement, Fourier transform infrared spectroscopy (FTIR), differential scanning calorimetry (DSC), and tensile mechanical testing. The physical features of nanoscaffolds were examined from multiple angles and the potential application of blended nanofibers in bone tissue engineering was assessed. The experimental results also provided theoretical evidence for the further application of minor ampullate spidroins in bone tissue engineering nanoscaffolds.

## 2. Results and Discussion

### 2.1. Structures of Proteins

**Figure 1:**
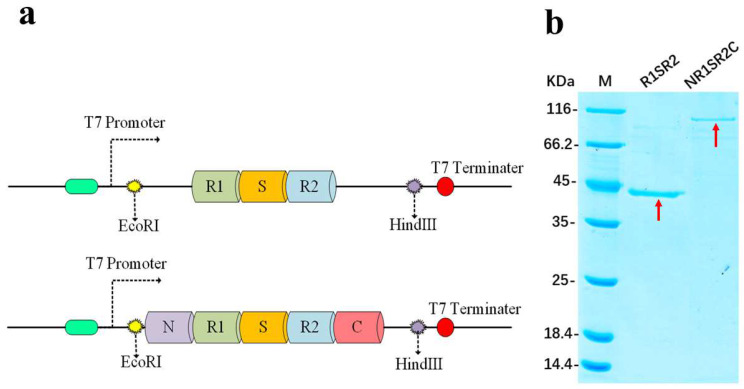
Production and purity of recombinant silk proteins. (**a**) Constructs. (**b**) SDS-PAGE results. The red arrows are bands of the target proteins.

Spidroins were composed of repeat units (R1 and R2), spacer units (S), and N-(N) and C-(C) termini of *Araneus ventricosus* MiSp, referred to as R1SR2 and NR1SR2C, respectively (Figure 1a). The R1, S, R2, N and C domains were 147, 127, 112, 160, and 146 amino acid-long, respectively (Supplementary Figure S1).

No previous study has examined the influence of N- and C-termini on the characteristics of fibers during electrospinning. Herein, we selected recombinant MiSp containing N- and C-termini or not for comparisons, since during the polymerization of spidroins are controlled simultaneously by these two structures [3]. The spider protein is expressed and accumulates in inclusion bodies. In this study, the denaturation-renaturation method was used for purification. SDS-PAGE confirmed accurate molecular size and purity for the spider protein obtained (Figure 1b). The purified proteins were separated by 12% SDS-PAGE. Image analysis revealed purity higher than 90% for the target proteins. The molecular weights of RISR2 and NR1SR2 were about 43 kDa and 85 kDa, respectively.

### 2.2. Morphology of Nanofibrous Scaffolds

Two recombinant spidroins were produced and blended with PLGA. We fabricated pure PLGA and spidroins (NR1SR2C and R1SR2)/PLGA electrospinning nanofibrous scaffolds with a weight ratio of 20/80 and analyzed their mechanical properties, respectively. The spinning process of R1SR2/PLGA (20/80) and NR1SR2C/PLGA (20/80) polymer solutions were the same, continuous and uniform, as that used for pure PLGA. We also fabricated blend nanofibrous scaffolds at different weight ratios to identify an ideal mix ratio of PLGA for electrospinning. However, it was hard to generate uniform nanofibers with spidroins (NR1SR2C and R1SR2) and PLGA mixed at weight ratios of 50/50 and 80/20. Therefore, all spidroins utilized here were blended with PLGA at 20/80 for the fabrication of electrospinning blend nanofibrous scaffolds.

**Figure 2:**
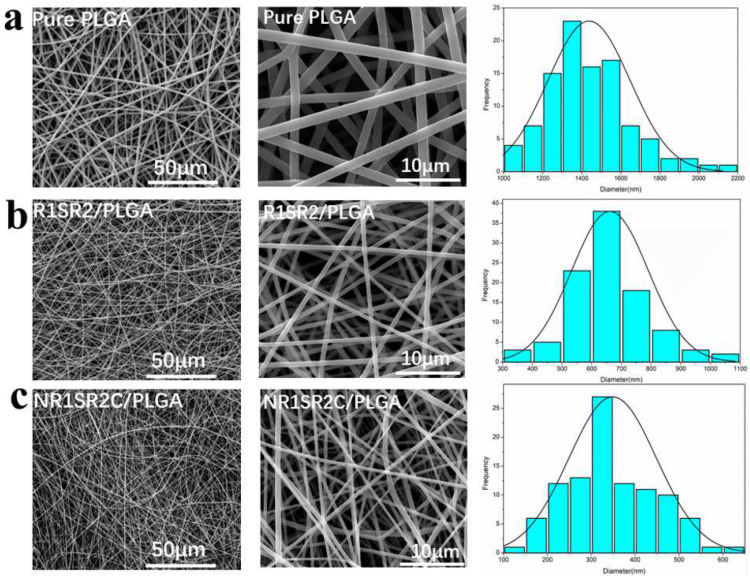
SEM scans and diameters of nanofibrous membranes. (**a**) Pure PLGA (**b**) R1SR2/PLGA (20/80) (**c**) NR1SR2C/PLGA (20/80).

Nanofibrous scaffolds were generated with 2–5 mL 10% (*w*/*v*) (NR1SR2C, R1SR2)/PLGA (20/80) spinning dopes by routine electrospinning methods. Pure PLGA nanofibrous scaffolds were also prepared in the same process for comparison. The surface of all types of scaffolds were examined by SEM (Figure 2). Every scaffold was composed of randomly oriented three-dimensional structures of the electrospun fibers and showed a porous structure.

The surface of all fibers was very smooth and uniform. The average diameter of the produced scaffolds was analyzed with Image J. The average nanofiber diameters of NR1SR2C/PLGA (20/80) and R1SR2/PLGA (20/80) blend scaffolds were about 300 nm to 700 nm (Table 1), which were significantly reduced compared with those of pure PLGA nanofibers (1437 ± 211 nm). The average diameter of NR1SR2C/PLGA (20/80) blend scaffolds was about half that of RSR/PLGA (20/80) blend scaffolds. However, R1SR2/PLGA (20/80) and NR1SR2C/PLGA (20/80) blend scaffolds possessed significantly higher porosity (75–86%) compared with pure PLGA scaffolds (56.7 ± 7.9%). The above findings suggest spidroins decrease the diameters of nanofibers and increase the porosity of scaffolds. Specifically, the N- and C-termini of spider silk protein significantly reduced the diameter of electrospun fibers and increased the porosity of blended scaffolds. A recent study demonstrated that the internal structure of hard outer layer of bone structure (spongy bone) is arranged in trabeculae with a porosity of 75–85% [45]. After adding the recombinant spidroins of minor ampullate, the porosity of the blend scaffold was surprisingly consistent with that of the scaffold, suggesting this material could well-simulate the primary bone environment when applied in bone tissue engineering scaffolds. For bone tissue co-engineering, the superior mechanical properties of the blend scaffold are extremely important. 

The images in the middle column show magnified versions of the scaffolds. This appeared at the images in the middle column (multiples 5000), showing that the average diameters of the fibers gradually decreased as spidroins blended in. After blending spidroins into PLGA, the diameter decrease of the blend scaffold is generally caused by elevated electrical conductivity of the mixed solution [46]. When PLGA is mixed with spider silk protein, the charge density of the solution is increased compared with that of pure PLGA. At this time, the electrostatic force has a stronger stretching effect on the jet, so a scaffold with lower fiber diameter is spun out. Reports have shown that the diameter of a blended fiber does not change with increases of repetition area and molecular weight [47]. Therefore, here we explored the influence of N/C terminal on fiber diameter. Previous studies based on magnetic resonance spectroscopy showed that the N terminal of MiSp is folded into a five-helical bundle, and the C-terminal of MiSp is a dimer of two five-helical bundles [3,48]. Although the N- and C-termini of most spider silk proteins are highly conserved and the C-terminal dimer contains disulfide bonds, the C-terminal of the MiSp protein used in this study does not contain cysteine [49], but still can form a stable secondary structure, indicating that the module contains amino acid residues with strong hydrophobicity [3,50], which could help reorder the structure of the repeating region, thus affecting fiber diameter in the process of electrospinning.

### 2.3. Structures

**Figure 3:**
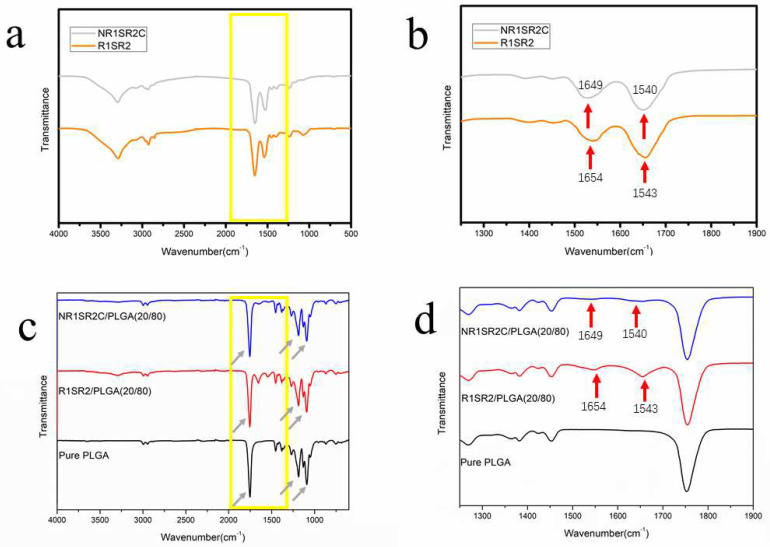
FTIR spectra of recombinant MiSp (NR1SR2C, R1SR2)/PLGA blend scaffolds and pure PLGA scaffolds. Peaks at 1089, 1187 and 1754 cm^−1^ indicated by arrows in (**c**) correspond to PLGA. (**b**,**d**), Zoomed-in image of (**a**,**c**) which in yellow box, from 1250 cm^−1^ to 1900 cm^−1^; red arrows indicate typical peaks of spidroins’ amide I bands (1600–1700 cm^−1^).

FTIR results are depicted in Figure 3. FTIR spectroscopy was employed for assessing the chemical compositions of pure spidroins(R1SR2,NR1SR2), pure PLGA scaffolds and spidroins (NR1SR2C, R1SR2)/PLGA (20/80) blend scaffolds. FTIR spectra of pure PLGA scaffolds and (NR1SR2C/R1S2R)/PLGA (20/80) blend scaffolds had characteristic absorption peaks at ~1089 and ~1187 cm^−1^ representing esters groups (C-O), as well as a typical carbonyl (C=O) peak at ~1754 cm^−1^, which are typical PLGA peaks [51] (Figure 3c). In addition, the blend scaffolds presented typical amide absorption peaks (N-H), including in the ranges of 1540–1543 and 1649–1654 cm^−1^ for amide II and I bonds, respectively. The blend scaffolds had typical absorption peaks of spidroins, consistent with the peaks of pure NR1SR2C (R1SR2) scaffolds in amide I bands (Figure 3d). The above data suggested spidroins in scaffolds displayed an α-helix/β-sheet mixed conformation [51,52,53]. Since blend scaffold spectra had peaks for both PLGA and spidroins, it can be inferred blend scaffolds were a mixture of spidroins and PLGA, which did not significantly interact.

**Figure 4:**
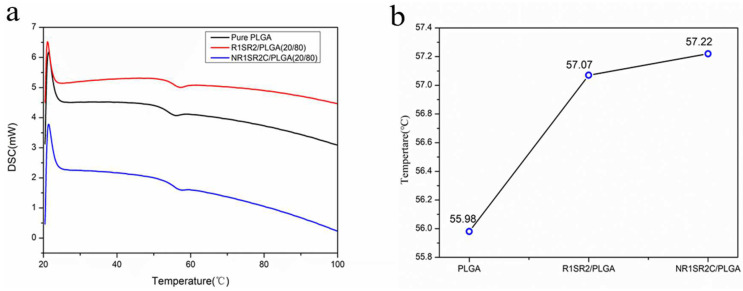
Thermal properties of the produced electrospun spidroins (NR1SR2C and R1SR2)/PLGA blend scaffolds and pure PLGA scaffolds. (**a**) DSC curves. (**b**) Tg values obtained from (**a**).

The thermal properties of the electrospun fibers were also determined. DSC data and Tg values are shown in Figure 4, which demonstrates that introducing spidroins (NR1SR2C, R1SR2) caused its Tg (glass transition temperature) to rise successively. Tg increased from 55.98 °C to 57.07 °C and 57.22 °C, respectively. The results showed a single glass transition temperature, indicating that the material mixing texture is uniform, and spider silk could improve the glass transition temperature of the blend scaffold. The increase of glass transition temperature may decrease the chain migration rate, to effectively prevent shrinkage, which is consistent with the reported performance of Misp that does not over-shrink in the presence of water [44].

### 2.4. Mechanical Properties

**Figure 5:**
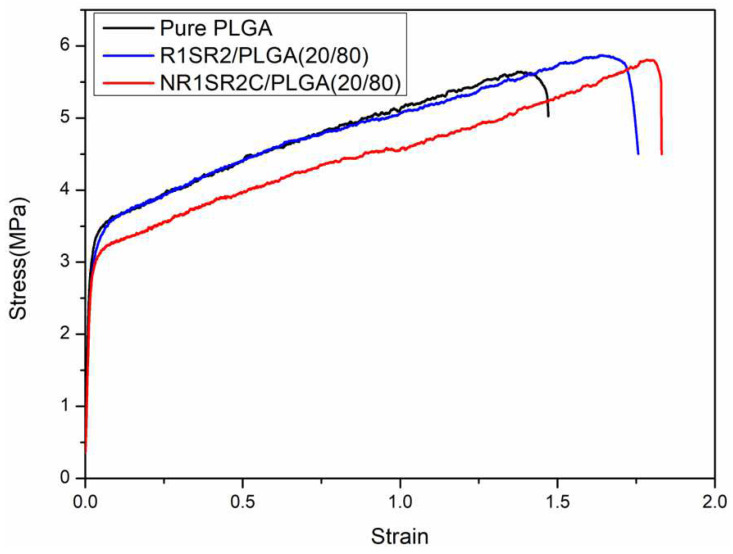
Representative stress–strain curves for recombinant MiSp/PLGA (20/80) blend and pure PLGA scaffolds.

The mechanical features of scaffolds are a critical factor for tissue engineering. Figure 5 shows representative stress–strain curves for R1SR2/PLGA (20/80) blend scaffolds, NR1SR2C/PLGA (20/80) blend scaffolds and pure PLGA nanofibrous scaffolds. The ultimate strain and stress of scaffolds were improved with the addition of spider silk proteins (spidroins). The ultimate tensile strength of NR1SR2C/PLGA (20/80) scaffolds was 5.81 MPa, which was comparable to that of R1SR2/PLGA (20/80) scaffolds (5.88 MPa) and superior to that of pure PLGA scaffolds (5.64 MPa). NR1SR2C/PLGA (20/80) scaffolds showed the most substantial elongation (183.41%). The ultimate elongation of R1SR2/PLGA (20/80) scaffolds was 173.21%, was much higher than that of pure PLGA scaffolds (146.88%). With the addition of spidroins, we speculated that the blending of a small amount of protein might act as a link in the blended scaffold. The protein’s structure might determine the length of the chain and the stretchable limit. Compared with pure PLGA, the addition of spidroins made some smaller stretching space reserved during the deformation process, thus increasing the ultimate strain of the elongation in the blended scaffolds.NR1SR2C/PLGA (20/80) scaffolds, with tensile modulus (150.1 ± 28.2 MPa), were the highest tensile modulus among the three examined nanoscaffolds. However, the tensile modulus of R1SR2/PLGA (20/80) scaffolds (127.6 ± 21.7 MPa) was close to that of pure PLGA (123.1 ± 17.6 MPa) (Table 2.). The data show that the mechanical features of all blend scaffolds are slightly decreased; however, the ultimate strain obviously improved compared with that of pure PLGA scaffolds. It may affect the rearrangement of molecules in the deformation process in the presence of the N- and C-termini of MiSp. Meanwhile, the decrease elastic limit and a different deformation pattern within the plastic region may be due to different interfacial properties between the spidroins and the polymer. In the present study, with the addition of recombinant MiSp, the mechanical properties of blend nanoscaffolds had a higher ultimate stress and tensile modulus than those of other composite PLGA blends the previous article reported [16]. 

### 2.5. Hydrophilicity of Nanofibrous Scaffolds

**Figure 6:**
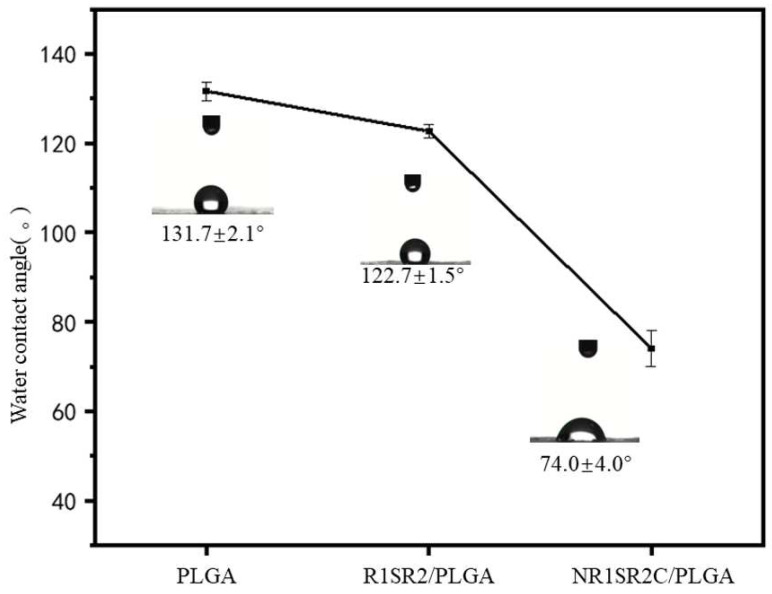
Hydrophilicity of recombinant MiSp/PLGA (20/80) blend and pure PLGA scaffolds (*n* = 3).

Cell adhesion and growth on biomaterial strongly depend on surface hydrophilicity, or sometimes termed wettability [54,55]. Therefore, the water contact angles of the designed scaffolds were measured for determining the effect of spidroins on surface hydrophilicity. Figure 6 shows a water contact angle for pure PLGA scaffolds of 131.7 ± 2.1°, which showed the highest value among pure PLGA scaffold and two blended scaffolds. The result illustrated that pure PLGA scaffolds are hydrophobic. R1SR2/PLGA (20/80) blend scaffolds had slightly reduced contact angle (122.7 ± 1.5°) than pure PLGA ones. Notably, NR1SR2C/PLGA (20/80) blend scaffolds had a significantly smaller contact angle, which was drastically decreased to 74.0 ± 4.0°. This finding demonstrated that with the N- and C-termini of spider silk protein, the contact angle of spidroins/PLGA blend scaffolds is reduced, suggesting the hydrophobic surface of the scaffolds has become hydrophilic. According to the previous research results, our material has appropriate hydrophilicity, indicating that it provides an environment for cell growth.

### 2.6. Biocompatibility of Nanofibrous Scaffolds

**Figure 7:**
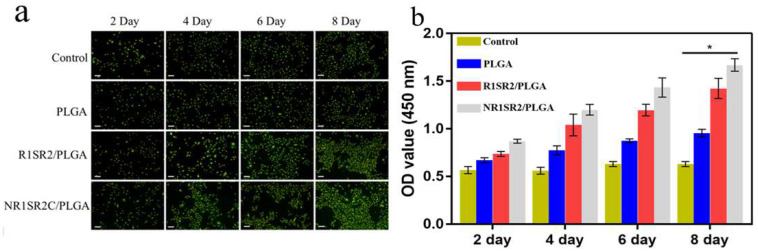
Fluorescent images (**a**) and quantitation (**b**) of HBMSC proliferation on recombinant MiSp/PLGA (28/80) blend and pure PLGA scaffolds (Scale bar: 400 μm, *n* = 3, * *p* < 0.05.).

Cell adhesion and proliferation on scaffolds represents the key factor in tissue engineering and clinical applications. However, cell adhesion and proliferation are highly dependent on the hydrophilicity of nanomaterials. Water contact angles for scaffolds PLGA and R1SR2/PLGA (20/80) were all greater than 90°, showing hydrophobic characteristics, while the water contact angle of scaffolds NR1SR2C/PLGA (20/80) was about 75°, which could provide a more suitable wettable environment for cell growth. Cell culture experiments were utilized to confirm this finding. BMSCs were cultured using hydrophobic scaffolds RISR2/PLGA (20/80) and hydrophilic scaffolds NRISR2C/PLGA (20/80) as substrates. Pure PLGA scaffolds were also implanted into cells for comparative assessments. To evaluate the adhesive and proliferation abilities of BMSCs on blended scaffolds, the activity of HBMSCs on different stents was evaluated by the CCK-8 assay and phalloidin staining, after 2, 4, 6, and 8 days of culture (Figure 7a,b). Adherent growth of HBMSCs was observed in all three substrates. On day 2, absorbance values were elevated for NR1SR2C/PLGA (20/80) and R1SR2/PLGA(20/80) blend scaffolds compared with other samples, while the absorbance values of the control group and pure PLGA scaffolds were basically unchanged, indicating that HBMSCS were more active on spider silk/PLGA(20/80) blend scaffolds compared with pure PLGA scaffolds. From day 4, the spider silk protein/PLGA material showed enhanced cell proliferation than pure PLGA. On days 6 and 8, the numbers of live cells on the NRSRC/PLGA (20/80) scaffold increased significantly. These results support a high proliferation of HBMSCs on NR1SR2C/PLGA (20/80) scaffolds.

**Figure 8:**
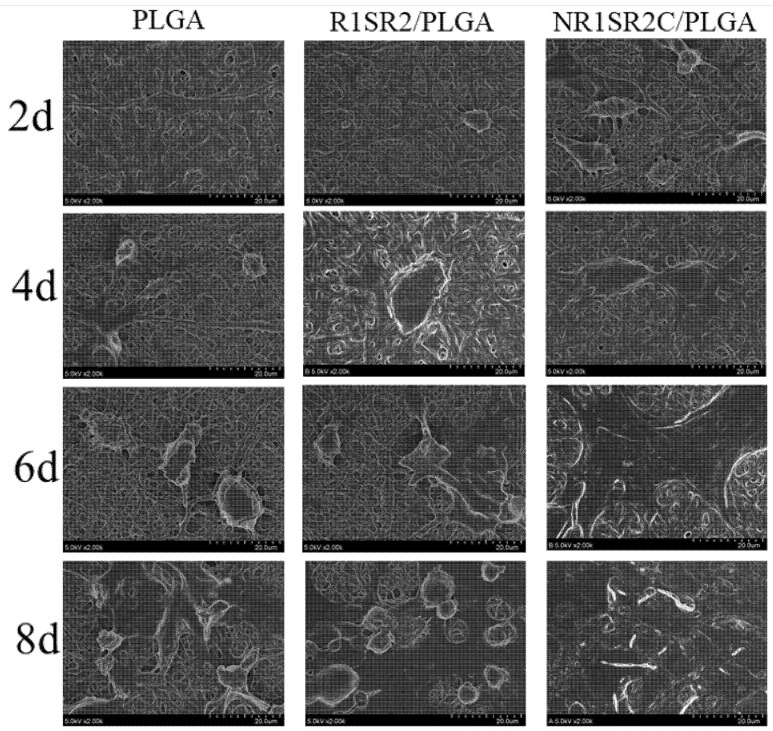
SEM scans of HBMSCs grown on recombinant MiSp/PLGA (20/80) blend and pure PLGA scaffolds for 2–8 days. Scale bar: 20 μm.

Cell proliferation and adhesion on the fibers were examined by scanning electron microscopy. As depicted in Figure 8, cells grew on the three scaffolds, with firm attachment and dispersion. The cells were fusiform or flat. With increasing culture time, cell diffusion became more obvious, with rising number and size. These results indicated that all scaffolds were harmless to cells and could provide biological microenvironments and physical support for cell growth. Scaffolds mixed with spider silk proteins showed superior cell growth and adhesion over pure PLGA scaffolds. From the sixth day, the cell paving area on the NR1SR2C scaffold was large, indicating that the cells were evenly distributed and proliferated well (Figure 8). By the eighth day, the cells had spread all over the field of vision. These results demonstrated HBMSCs were widely distributed and had the highest density in NR1S2RC/PLGA (20/80) blended scaffolds. SEM data for these cells also supported the growth trend in the above fluorescent images, suggesting that the addition of spider silk protein could improve cell adhesion and proliferation. Cell growth also showed a positive correlation with the improvement of hydrophilicity of the blend scaffold.

## 3. Materials and Methods

### 3.1. Materials

1, 1, 1, 3, 3, 3 hexafluoro 2-propanol (HFIP) was provided by Shanghai Darui Finechemical (Shanghai, China). Poly(lactic-co-glycolic) acid (PLGA, molar ratio of PLA: PGA = 75:25) was provided by Jinan Daigang Biomaterial (Jinan, China). Competent cells, chemicals and kits were provided by Sangon Biotech (Shanghai, China). Restriction enzymes were provided by Thermo scientific (Waltham, MA, USA). Phusion PCR Master Mix and T4 ligase were provided by New England Biolabs (Beijing, China). Human bone mesenchymal stem cells (HBMSCs) were provided by Shanghai Institute of Biochemistry and Cell Biology (SIBCB, CAS, Shanghai, China). They are commercialized cell lines. Dimethyl sulfoxide (DMSO) was provided by Changshu Hong sheng Chemical Reagent (Suzhou, China). Paraformaldehyde (POM) and Cell Counting Kit -8 (CCK-8) were provided by Biyotime Institute of Biotechnology (Haimen, China). Dulbecco’s Modified Eagle’s Medium (DMEM), fetal bovine serum (FBS) and phosphate-buffered saline (PBS) were provided by Mesgen Company (Shanghai, China). Penicillin/streptomycin and trypsin were provided by Shanghai Yuanxiang Medical Equipment (Shanghai, China). A Milli-Q Plus 185 water purification system (Millipore, Burlington, MA, USA) was utilized to produce ultrapure water (18 MΩ cm). 

### 3.2. Plasmid Construction

Plasmids harboring a recombinant spidroin with a His-tag and a single repeat unit (R1), a spacer unit (S) and another repeat unit (R2) of *Araneus ventricosus* minor ampullate spidroin (MiSp) (GenBank accession number, JX513956) were constructed. For constructing another plasmid NR1SR2C harboring the MiSp, N- and C-termini also from MiSp were added to both ends of R1SR2, respectively, utilizing polymerase chain reaction (PCR), restriction enzyme cleavage and DNA ligation. Nucleotide sequencing was carried out for verifying sequence accuracy.

### 3.3. Protein Production

Escherichia coli BL21 (DE3) cells (Tiangen, China) transformed with various plasmids underwent culture in an incubator at 37 °C with shaking at 220 rpm in Luria broth with 50 μg/mL ampicillin. At OD600 of 0.6–0.9, bacteria were treated with isopropyl β-D-1-thiogalactopyranoside (IPTG) at 0.3 mM, followed by incubation at 16 °C and 180 rpm for 16 h. Upon cell harvesting by a 15 min centrifugation (4 °C, 4000 rpm), lysis was performed with lysis buffer (20 mM Tris-HCl, 100 mM NaCl, pH 8.0) using a JN-3000 plus high-pressure homogenizer (JNBIO, Guangzhou, China) at 1200 bar. The target proteins in the pellet upon a 40 min centrifugation (4000 g, 4 °C) were re-suspended in washing buffer (2–4 M urea, 20 mM Tris-HCl, pH 8.0, 100 mM NaCl), followed by sonication for 30 min at 250 W (5 s ON and 8 s OFF) in an ice bath. After another 40 min centrifugation (4000 g, 4 °C), the resulting pellets were resolubilized in a dissolution buffer (8 M urea, 20 mM Tris-HCl, pH 8.0, 100 mM NaCl, and 100 mM β-ME). The sample was sonicated again and incubated at ambient for 30 min. Insoluble materials were cleared by a 30 min centrifugation (12,000 rpm, 25 °C). The protein solution next underwent gradient-dialysis at 4 °C for 4 h against dialysis buffers (20 mM Tris-HCl, pH 8.0, 100 mM NaCl) containing 6, 4, and 2 M urea, respectively. Upon de-ionization in water for 72 h, the dialysate was lyophilized on a lyophilizer (Labconco, Kansas City, MO, USA) and assessed by SDS-PAGE and Coomassie Brilliant Blue staining for purity evaluation.

### 3.4. Preparation of Synthetic Spider-Silk Fibers

Purified spidroin solutions underwent dialysis against deionized water at 4 °C overnight and lyophilization for the pulverization of spidroins. Spidroin powders and PLGA were blended (28/80, *w*/*w*) and mixed with pure 1,1,1,3,3,3-hexafluoro-2-propanol (HFIP) at 10% (*w*/*v*). Spinning dopes were then stirred for 24 h at ambient. 

**Figure 9:**
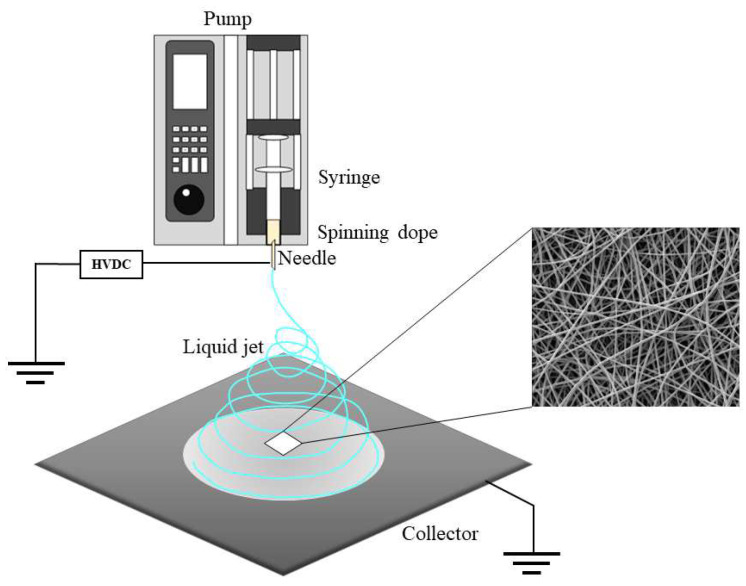
Schematic representation of the electrospinning process. HVDC, high-voltage direct current.

Recombinant MiSp (NR1SR2C, R1SR2)/PLGA (20/80) blend nanofibrous scaffolds were fabricated with a routine electrospinning protocol. Spinning dopes were loaded into a 2 or 5 mL syringe with a 23G stainless steel spinneret and ejected at 0.6 mL/h by a LSP01-3A syringe pump (Longer Precision Pump Co. Ltd., Shanghai, China). The voltage was 12 kV, and collection distance (the vertical distance from the spinneret end to the collector) approximately 15 cm. Pure PLGA nanoscaffolds were also electrospun under identical conditions. The procedures are summarized in Figure 9. Upon electrospinning, the nanofibrous scaffolds underwent drying in a vacuum desiccator for 24 h prior to subsequent assays at ambient.

### 3.5. Scanning Electron Microscope (SEM) of Nanofibrous Scaffolds

The nanofibrous scaffolds were surface-coated with Au particles for 45 s and analyzed under a SEM (Phenom-World BV, Eindhoven, The Netherlands) at 10 kV. Diameter distribution for the fibers was assessed with ImageJ [56] and >100 nanofibers on SEM scans were randomly assessed for various samples.

### 3.6. Fourier Transform Infrared (FTIR) Spectroscopy 

A Nicolet 6700 FITR spectrometer (Thermo Fisher) was utilized for recording spectra for nanofibrous scaffolds from 4000 to 600 cm^−1^ in the attenuated total reflection mode at ambient. For every spectrum, 64 scans were averaged at a resolution of 1 cm^−1^. Amide I regions (1600–1700 cm^−1^) of every specimen were next assessed for identifying the peaks of α-helix and β-sheet structures in spidroins.

### 3.7. Differential Scanning Calorimetry (DSC)

DSC was performed on a TA instrument (204F1 NetZSch, Selb, Germany) for determining the thermal features of electrospun nanofibrous scaffolds, with Tg derived as previously described [57].

### 3.8. Water Contact Angle Measurements

The water contact angles of scaffolds were obtained via a drop-shape analyzer DSA30 (KRUSS, Hamburg, Germany) using the sessile drop method. Data analysis was performed with ADVANCE-drop shape for determining contact angles. A minimum of 3 distilled water drops in various positions were assessed per sample.

### 3.9. Mechanical Testing 

Nanofibrous scaffolds were cut at 20 mm × 10 mm for mechanical property measurements. Sample thickness was assessed by a screw micrometer and approximated 10–30 μm. A universal material tester H5K-S (Hounsfield, UK) was utilized for assessing the specimens at ambient with a cross-head rate of 20 mm/min. Average mechanical features for various scaffolds were obtained from a minimum of 6 specimens.

### 3.10. Cell Culture on Nanofibrous Scaffolds

Prior to in vitro assays, HBMSCs underwent culture in RPMI-1640 containing 10% fetal bovine serum (FBS) and 1% penicillin/streptomycin at 37 °C with 5% CO_2_. Round electrospinning nanofibrous scaffolds (14 mm in diameter) were prepared as reported previously [58]. Briefly, scaffolds were generated on circular glass cover slips, followed by sterilization with 75% ethanol for 2 days and ultraviolet irradiation for 1–2 h. Then, the scaffolds were placed in 24-well plates and underwent fixation with stainless rings. In proliferation and adhesion assays, HBMSCs underwent seeding on the produced scaffolds in 24-well plates at 3 × 10^4^ cells/well and culture for 2, 4, 6 and 8 days at 37 °C with 5% CO_2_, respectively, with the culture medium refreshed at 48 h intervals.

### 3.11. Cell Proliferation and Attachment Assays

Round electrospinning nanofibrous scaffolds (14 mm in diameter) were produced as reported previously [34] and placed in 24-well plates. To assess HBMSC proliferation and adhesion, HBMSCs underwent seeding on scaffolds in 24-well plates at 3 × 10^4^ cells/well, followed by culture for 2, 4, 6 and 8 days at 37 °C with 5% CO_2_, with the culture medium refreshed at 48 h intervals [44]. Next, scaffolds underwent PBS washes and staining with phalloidin for 20 min at ambient shielded from light. A fluorescence microscope (Olympus, BX53, Tokyo, Japan) was used for analysis. Additionally, to examine HBMSC proliferation and attachment on nanofibrous scaffolds, the seeded scaffolds underwent three PBS washes and fixation with 4% paraformaldehyde overnight. This was followed by dehydration with graded ethanol (30%, 50%, 70%, 80%, 90%, 95% and 100%) and drying under vacuum. Finally, the scaffolds were surface-coated with Au particles prior to SEM.

### 3.12. Statistical Analysis

Analysis of variance (ANOVA) was carried out using Origin 8.5 (OriginLab, Inc., Northampton, MA, USA) to determine whether at least two data sets showed differences. *p* < 0.05 indicated statistical significance.

## 4. Conclusions

In this study, recombinant MiSp and PLGA were blended at a ratio of 20/80 to generate a uniform nanoscaffold, which was utilized to evaluate the application potential of the recombinant MiSp/PLGA blended scaffold in bone tissue engineering. This was the first study blending recombinant MiSp with PLGA by electrospinning.

The results showed blending the scaffold with recombinant MiSp could improve the mechanical features of the material, especially its ductility. Meanwhile, recombinant MiSp increased the hydrophilicity of the blend scaffold. It caused the water contact angle to decrease from 131.7 ± 2.1° to 74.0 ± 4.0° and slightly increased the Tg temperature of the material from 55.98 °C to 57.22 °C. With the addition of N- and C-termini, part of material performance optimization becomes more significant. Among them, NR1SR2C blend nanoscaffolds were superior to all nanoscaffolds in terms of performance.

The average diameter of nanoscaffolds decreased with the addition of recombinant MiSp, 1437 ± 211 nm for pure PLGA to 349 ± 100 nm. It is worth noting that the average diameter of the recombinant Misp containing the N- and C-termini of the minor ampullate was reduced by nearly half (349 ± 100 nm) compared with that without the C- and N-terminal nanoscaffolds R1SR2/PLGA (20/80) of 661 ± 130 nm. NR1SR2C/PLGA nanofibers scaffolds have sizes ranging from 50–500 nm; therefore, the fibers sizes are similar to that of nanofibrous collagen (a nature extracellular matrix). In the present study, with the addition of recombinant MiSp, the mechanical properties of blend nanoscaffolds had a higher ultimate stress and tensile modulus than those of other composite PLGA blends the previous article reported [16]

In vitro, the blended scaffold showed enhanced adhesion compared with pure PLGA and significantly induced cell proliferation. This advantage was most significant in the NR1SR2C blended scaffold, which may be associated with the improved hydrophilicity of the material.

Meanwhile, the above results revealed that the blend nanoscaffold with the recombinant MiSp with the N- and C-termini could significantly improve the hydrophilicity of the material, and suitable hydrophilicity compared with the hydrophobic structure of pure PLGA could provide a more appropriate environment for cell growth and proliferation.

According to the above results, we produced blend scaffolds with potential properties and suitable for bone tissue engineering research. Additionally, the results predicted that the N- and C-termini of the MiSp could be utilized as functional modules to conjugate with other proteins to adjust the diameter, hydrophilicity and porosity of the blend nanoscaffolds. There are active groups on the surface of spider silk protein, which is easy for secondary modification [46], also providing a basis for further optimization of properties to obtain materials suitable for bone tissue engineering in the future.

These results indicate that the blend nanoscaffolds made from the recombinant MiSp/PLGA are potential materials for bone tissue engineering.

## Figures and Tables

**Table 1:** Mean fiber diameters and porosity of R1SR2/PLGA (20/80) and NR1SR2C/PLGA (20/80) blend scaffolds and pure PLGA scaffolds.

Scaffold Type	Diameter (nm)	Porosity (%)
PLGA	1437 ± 211 nm	56.7 ± 7.9%
R1SR2/PLGA (20/80)	661 ± 130 nm	75.5 ± 3.7%
NR1SR2C/PLGA (20/80)	349 ± 100 nm	85.9 ± 6.1%

**Table 2:** Mechanical features of nanofibrous membranes (6 times per membrane).

Scaffold Type	Stress (MPa)	Strain (%)	Tensile Modulus (MPa)
PLGA	5.4 ± 0.5	142.3 ± 10.5	123.1 ± 17.6
R1SR2/PLGA (20/80)	5.8 ± 0.8	170.9 ± 8.4	127.6 ± 21.7
NR1SR2C/PLGA (20/80)	5.8 ± 1.3	181.6 ± 18.7	150.1 ± 28.2

## Data Availability

Not applicable.

## Supplementary Materials

The following supporting information can be downloaded at: https://www.mdpi.com/article/10.3390/ijms24021219/s1.

## Abbreviations

MiSp	Minor ampullate spidroins
PLGA	Poly(lactic-co-glycolic) Acid
SEM	Scanning electron microscopy
FTIR	Fourier transform infrared spectroscopy
DSC	Differential scanning calorimetry
HBMSCs	Human bone mesenchymal stem cells
