# Supplementary material for: Characteristic Evaluation of Recombinant MiSp/Poly(lactic-co-glycolic) Acid (PLGA) Nanofiber Scaffolds as Potential Scaffolds for Bone Tissue Engineering
Source: Int J Mol Sci. 2023 Jan 7;24(2):1219. doi: 10.3390/ijms24021219 (PMC9861889; doi:10.3390/ijms24021219)
Supplement: Supplementary file 1 [file ijms-24-01219-s001.zip › ijms-2108203-supplementary.pdf]

Supplementary information

Evaluation of the application of recombinant MiSp/Poly(lactic-co-glycolic) Acid (PLGA) Nanofibrous Scaffolds for Bone Tissue Engineering

Yuan Sun, Xiaona Jia and Qing Meng*

College of Biological Science and Medical Engineering, Donghua University, Shanghai 201620, China;
1152060@mail.dhu.edu.cn (Y.S); xiaonajia@126.com (X.J)

* Correspondence: mengqing@dhu.edu.cn

N:
CAACCAATCTGGACCAACCCAAATGCAGCAATGACCATGACCAACAACCTGGTCCAATGTGCGAGTCGGTCAGGTGTGCTC
ACAGCCGATCAGATGGACGACATGGGAATGATGGCAGACTCTGTTAACTCGCAGATGCAGAAAATGGGACCAAACCCACCT
CAACACAGACTCAGGGCAATGAATACCGCCATGGCCGCAGAAGTAGCTGAAGTAGTAGCAACTTCGCCACCACAAAGTTAT
TCTGCAGTTTTAAATACCATTGGTGCTTGCTTGAGGGAATCAATGATGCAAGCGACAGGCTCCGTCGACAATGCATTCACAA
ATGAAGTAATGCAATTGGTAAAAATGTTATCTGCGGATAGCGCGAATGAAGTATCTACAGCAAGTGCATCAGGAGCCAGTT
ACGCAACAAGTACGTCATCTGCAGTAAGCTCATCTCAAGCAACAGGATACAGCACTGCAGCAGGTTATGGAAAC

R1:
GGAGAGGGAGCAGGTGCAGCAGCAGCGGCTGGTGCAGGAGCAGGAGGTGCAGGTGGATACGGAGGAGGAGCCGGCTCTGG
AGCTGGTGCGGTTGCTAGAGCAGGGGCGGGAGGTGCCGGAGGATACGGCTCAGGAATTGGCGGTGGATATGGATCGGGAGC
AGGAGCTGCGGCGGGTGCTGGAGCAGGAGGTGCAGGTGCGTATGGAGGAGGTTACGGTACTGGAGCCGGAGCAGGCGCAA
GAGGAGCGGATAGTGCAGGAGCTGCAGCTGGATACGGTGGAGGAGTTGGTACCGGAACTGGAAGTTCCGCAGGATACGGA
AGAGGAGCCGGTGCTGGAGCCGGAGCAGGTGCTGCAGCTGGATCAGGAGCTGGAGCTGCAGGGGGATATGGAGGAGGTTA
CGGTGCAGGTGCAGGTGCTGGCGCAGGAGCAGGAGGAGCT

S:
ACAGGCAACAGGGCTGGAGACGCATTCGCACAAGTATTCAGTCAGAATGTGATAAATTCTGGTGTTATCACTTCCACAACTG
TCACTAAAAATTCTGCACAAGCAGCTGCTTCTTCGATGGTGTCGACTGCTGCGAAGAGTCTCGGCTTGGATGAAAATACAGC
AAGAAGCATGGCTAATGCAATGTCCAGTTATGCTGCTGCTATGGCGAAATCTTTTAGAAACTCAGACGAATTTATCCGTAAC
ATGTCTTATCAAATGGGAAGGATGTTATCGAATGCCGGTGCGATTAATGAAAGCACAGCCTCGGCTGCTGCGTCGAGTGCTT
CTTCTACAGTAACAGAAACTGTAAGAACTTATGGACCTGCAGCAATCTTTAGT

R2:
GGAGCTGGTGCAGGAGGTGCCGGTGGATATGCCCAAGGCTATGGGGCTGGAGCTGGCGCTGGTGCTGGTGCAGGAACAGGA
GCAGGCGGCGCTGGAGGATATGGCCAAGGCTATGGTGCTGGATCAGGTGCAGGAGCTGGAGGTGCAGGAGGATATGGAGC
AGGAGCTGGTGCAGGAGCTGGAGCAGGAGGTGCCAGTGGATATGGACAAGGCTACGGCGATGGAGCTGGAGCAGGTGCTG
GAGCTGCAGCAGCGGCTGGTGCTGCTGCTGGAGCTAGAGGTGCAGGAGGATATGGAGGAGGAGCTGGTGATGGAGCAGGT
GCGGGAGCAGGAGCA

C:
ACTGTAGCTGCATATGGTGGCGCAGGTGGAGTTGCAACATCTTCAAGTTCGGCAACTGCCAGTGGATCTCGTATAGTTACAT
CTGGAGGTTACGGATATGGAACCAGTGCAGCTGCAGGAGCTGGAGTTGCAGCAGGTTCATATGCAGGTGCTGTCAATCGCTT
GTCTAGTGCTGAAGCTGCCAGTAGAGTATCCTCTAATATTGCAGCTATTGCATCTGGTGGTGCTTCCGCCCTCCCCAGTGTTA
TTTCAAATATTTACTCAGGTGTCGTTGCTTCTGGTGTTTCTTCTAATGAAGCTCTGATTCAAGCTCTGTTGGAACTCCTTTCCG
CACTTGTTCATGTTTTAAGCAGTGCCTCTATCGGTAATGTTAGCTCAGTAGGAGTAGATAGTACATTGAATGTTGTTCAGGAT
TCAGTAGGCCAATATGTAGGTTAA

Figure S1: The base sequences of the protein modules

N,R1,S,R2 and C units base sequences from the full-length template Araneus ventricosus minor ampullate spidroin (MiSp) (GenBank accession number, JX513956)."
